# Clonality, polyploidy and spatial population structure in Baltic Sea *Fucus vesiculosus*

**DOI:** 10.1002/ece3.9336

**Published:** 2022-09-20

**Authors:** Roxana Preston, Jaanika Blomster, Ellen Schagerström, Perttu Seppä

**Affiliations:** ^1^ Ecosystems and Environment Research Programme Faculty of Biological and Environmental Sciences University of Helsinki Helsinki Finland; ^2^ Tvärminne Zoological Station University of Helsinki Hanko Finland; ^3^ Department of Ecology, Environment and Plant Sciences Stockholm University Stockholm Sweden; ^4^ Stockholm University Baltic Sea Centre Stockholm University Stockholm Sweden; ^5^ Organismal and Evolutionary Biology Research Programme Faculty of Biological and Environmental Sciences University of Helsinki Helsinki Finland

**Keywords:** algae, clonal growth, gene flow, genetic diversity, mixed‐ploidy, reproductive mode

## Abstract

Genetic characteristics of populations can have substantial impacts on the adaptive potential of a species. Species are heterogeneous, often defined by variability at a range of scales including at the genetic, individual and population level. Using microsatellite genotyping, we characterize patterns underlying the genetic heterogeneity in marine macroalga *Fucus vesiculosus*, with a particular focus on two forms: attached and free‐living. Here we demonstrate that sympatric populations representing the two forms display marked differences in characteristics of reproduction and genetic diversity. Asexual reproduction was ubiquitous in the free‐living form despite being almost entirely absent in the attached form, while signals of polyploidy were common in both forms despite the distinct reproductive modes. Gene flow within and between the forms differed, with barriers to gene flow occurring between forms at various spatial scales due to the reproductive modes employed by individuals of each form. The divergent genetic characteristics of *F. vesiculosus* demonstrate that intraspecific differences can influence the properties of populations with consequential effects on the whole ecosystem. The differing genetic patterns and habitat requirements of the two forms define separate but closely associated ecological entities that will likely display divergent responses to future changes in environmental conditions.

## Graphical abstract

Using microsatellite genotyping we characterise patterns underlying the genetic heterogeneity in two forms of marine macroalga *Fucus vesiculosus*. Sympatric populations display marked differences in characteristics of reproduction, genetic diversity and population structure.

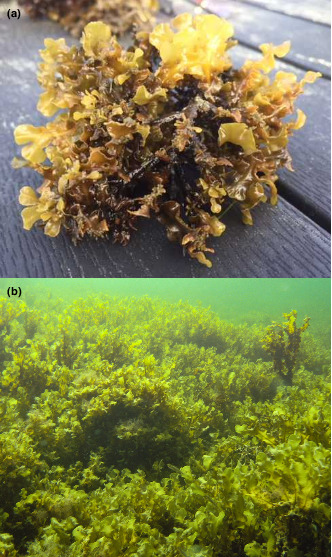

## 1 INTRODUCTION

Genetic diversity is vital for the adaptive potential of species, but it can also be important for whole ecosystem functioning (Zimmermann et al., 2012). It can have substantial ecological consequences at the population, community and ecosystem levels, being comparable to the effects of species diversity (Hughes et al., 2008). Spatial distribution of genetic diversity also represents information on evolutionary processes, including connectivity of populations, genetic drift, selection, and adaptation (Kitamura et al., 2018). Thus, factors affecting the genetic diversity and distribution of genetic diversity are key when species resilience to changing environmental conditions is assessed.

Reproductive mode (sexual vs. asexual reproduction) affects genetic diversity within populations and the amount of genetic differentiation among them (Ellegren & Galtier, 2016; Hamrigk & Godt, 1996). Moreover, reproductive mode can vary intraspecifically among populations, for instance, in plants (Johnson et al., 2020; Li et al., 2018) and algae (Loffler et al., 2018; Robitzch et al., 2019; Tatarenkov et al., 2005; Yamano et al., 2020). Both reproductive modes may also occur simultaneously within an individual (e.g. in plants [Vallejo‐Marín et al., 2010; Yang & Kim, 2016], animals [Braga‐Pereira & Santos, 2021; Lampert, 2008], and algae [Hawkes, 1990; Rafajlović et al., 2017]). Asexual reproduction has classically been misconceived as a factor that reduces genetic variation (Bengtsson, 2003), incurring genetic consequences similar to inbreeding in sexual populations, such as reduction in genetic diversity and inbreeding depression (Halkett et al., 2005; Vallejo‐Marín & Hiscock, 2016); however, this notion is contentious (Bengtsson, 2003; Ellstrand & Roose, 1987; Suomalainen et al., 1987). In facultatively asexual species, sexual reproduction can be limited due to biotic and abiotic aspects of the environment (Eckert, 2002). For example, salinity can influence allocation trade‐offs between reproductive modes in marine ecosystems (Dańko et al., 2020; Kostamo & Mäkinen, 2006; Lubzens et al., 1985).

Alongside the reproductive mode, polyploidization (whole‐genome multiplication) is an important variable affecting population divergence and gene flow (Brown & Young, 2000), and the interaction between asexual reproduction and polyploidization may affect genetic diversity and spatial genetic structure. Polyploidization often causes sterility as has been observed in angiosperms (Meichssner et al., 2021) and in some cases algae (Lewis & Neushul, 1995; Zhang & van der Meer, 1988). However, polyploids are often more vigorous compared to diploid conspecifics (Renny‐Byfield & Wendel, 2014) although polyploidization has been seen to pose little to no advantage in some algae (Patwary & van der Meer, 1984; van der Meer & Patwary, 1983; Zhang & van der Meer, 1988). Even if sterile, polyploidization can be favorable, at least in the short term, if linked to improved fitness traits because many polyploids are able to reproduce asexually (Comai, 2005) facilitating the rapid colonization and dominance of new areas (Lasker & Coffroth, 1999; Wulff, 1991). Thus, polyploids may have a competitive short‐term advantage, increasing their representation within the population.

**FIGURE 1:**
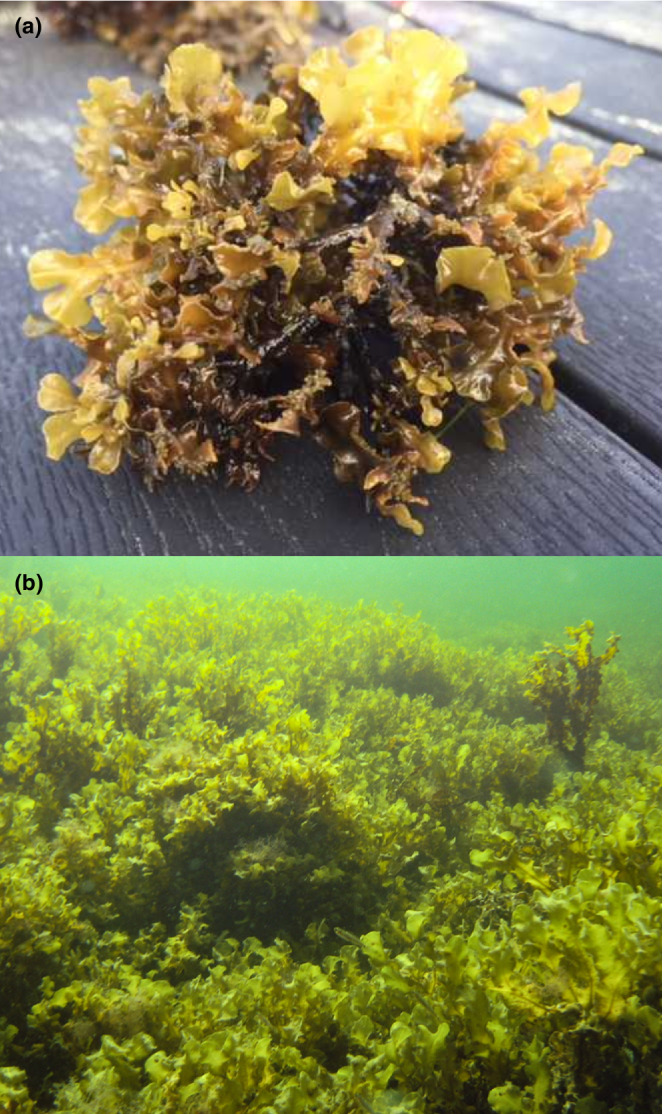
An image of an aegagropiloid free‐living *Fucus vesiculosus* thalli from Tvärminne, Finland (a) and a free‐living *F. vesiculosus* population at Askö, Sweden (b).

Here we investigate the genetic structure of the Baltic Sea population of the marine macroalga *Fucus vesiculosus*. *Fucus vesiculosus* is a perennial, dioecious and facultatively asexual species, which occurs at its range margin in the brackish Baltic Sea (Takolander et al., 2017). In the Baltic Sea, *F. vesiculosus* can be found in two forms, the most frequently studied being the epilithic form (hereby referred to as attached), but it is also found as a benthopleustophytic form (hereby referred to as free‐living; Figure 1) on any substrate within the photic zone (HELCOM, 2013). Traditionally, the free‐living form has been assumed to be entirely formed by asexual reproduction due to the absence (Svedelius, 1901) or sterility (Bauch, 1954; Häyrén, 1949) of receptacles, but this is based on anecdotal observations.

Asexual reproduction in attached Baltic Sea *F. vesiculosus* has been observed in both natural populations (Ardehed et al., 2016; Johannesson et al., 2011; Pereyra et al., 2013; Tatarenkov et al., 2005) and in laboratory experiments (Tatarenkov et al., 2005). Clonality (synonymous with asexual reproduction [De Meeûs et al., 2007 ]) has also been observed in several natural attached *Fucus* populations along the Finnish and Swedish coasts of the Bothnian Sea (Pereyra et al., 2009; Rinne et al., 2018), although the species identity of clonal samples (*F. vesiculosus* or *F. radicans*) has not always been clear. Although clonality within the attached Baltic Sea *F. vesiculosus* form is more commonly documented, unattached forms are also observed to display clonal growth. An embedded form of *F. vesiculosus* in the western Baltic Sea near Glücksburg (Germany) reproduces entirely by clonal growth (Meichssner et al., 2021). However, the mode of reproduction is poorly understood in free‐living *F. vesiculosus*. Nevertheless, the assumptions on the association between asexual reproduction and the free‐living form may have some validity.

Clonal growth complicates the definition of an individual, and thus here we briefly define the terminology. Genet refers to the entire clone or genotype, while ramet refers to the unit or module of clonal growth denoting the smallest physiologically integrated parts of a genet (Harper, 1977; Tuomi & Vuorisalo, 1989). Genet consequently infers a single clonal lineage in clonal thalli. As all parts of the *Fucus* thallus are photosynthetically active (Kremer, 1975), any module has the potential to became an independent ramet (Collado‐Vides, 2001).

Polyploidy is relatively poorly understood in Phaeophyceae (Bringloe et al., 2020) although it has been widely reported (Lewis, 1996; Neiva et al., 2017; Phillips et al., 2011; Ribera Siguan et al., 2011; Sousa et al., 2019; Yabu & Sanbonsuga, 1987; Yabu & Yasui, 1983). Polyploidization has been observed in several *Fucus* species in Spain (Gómez Garreta et al., 2010) and also in the Northern Atlantic, with some unattached forms being attributed to polyploid versions of the attached *Fucus* in the surrounding area (Coyer, Hoarau, Pearson, Serrão, Stam, Olsen, 2006; Sjøtun et al., 2017). To our knowledge, however, polyploidy has not been documented in the Baltic Sea *F. vesiculosus* and all previous genetic studies have assumed a diploid genetic system (e.g. Ardehed et al., 2016; Johannesson et al., 2011; Rinne et al., 2018).

In this work, we investigate the reproductive mode and spatial genetic structure in the Baltic Sea *F. vesiculosus* population by using DNA microsatellite markers. We focused on the two forms – attached and free living – sampling both forms from the same localities. We compared the amount of genetic variation in the two forms, determined the representation of reproductive modes (sexual vs. asexual reproduction in the form of clonal growth) in the study populations and assessed the spatial genetic structure across the study area. We hypothesized that the attached form reproduces primarily sexually and the free‐living form primarily asexually. We also hypothesized spatial structuring among all populations, although connectivity between sympatric populations was predicted to be relativity high. Finally, based on previous studies, we expected that polyploidy would be absent in the Baltic Sea *F. vesiculosus*, yet we also found a substantial proportion of potential polyploid samples in our study populations. This allowed us to consider the association between genome size and form.

## 2 MATERIALS AND METHODS

### 2.1 Study location and sample collection

*Fucus vesiculosus* thalli were sampled during 2017–2018 from 20 locations within the Baltic Sea along the Swedish coast of the Northern Baltic proper, the Finnish coasts of the Archipelago Sea and the Gulf of Finland, the Estonian coast of the Gulf of Riga, and the German coast of the Arkona basin (Appendix S1 and S2). Where possible both forms were sampled from the same site, however, on occasion only allopatric populations were available. Attached samples were taken from hard‐bottom substrata, whereas free‐living samples were from both soft and hard bottom substrata. Depth range for all sites varied between 0.5 and 3 m with free‐living and attached samples from the same localities taken at the most similar depth feasible. At each site 26–77 individual thalli per form type were randomly collected ensuring sufficient separation between samples depending on the size of thalli within the population (Appendix S2). The total number of samples collected was 1447, with the final analyzed number totalling 1443. Depending on depth samples were collected by SCUBA, snorkeling or wading. The thalli were cleaned of epiphytes and stored in silica gel prior to DNA extraction.

### 2.2 DNA extraction and microsatellite genotyping

Genomic DNA was extracted from 4 mg of dried apical tips using NucleoSpin® plant II DNA extraction kit (Machery‐Nagel, 740770.250) following the standard kit protocol and PL1 buffer for cell lysis. Eight polymorphic microsatellite loci – L20, L38, L58, L85, L94 (Engel et al., 2003), FSP1, FSP2, FSP3 (Perrin et al., 2007) – were targeted (Appendix S3). PCR reactions were performed using OneTaq® 2× Master Mix with Standard Buffer (New England Biolabs, M0482L) or OneTaq® Hot Start 2× Master Mix with Standard Buffer (New England Biolabs, M0484L). The full genotyping protocol is provided in the Appendix. Samples were genotyped on the ABI 3730 DNA analyzer in the Molecular Ecology and Systematics (MES) laboratory at the University of Helsinki.

### 2.3 Data analysis

Alleles were scored using Genemapper 5 (Applied Biosystems™) and checked by eye. A sizable proportion of individuals displayed more than two alleles in at least one of the loci studied. Thorough validity checks were performed including repeat DNA extractions, PCR reactions, and ABI plate setups with new reagents to determine the validity of the third and/or fourth allele. The trend of ≥3 observed alleles in the electropherograms was consistent and repeatable for the apparent polyploid specimens. Allele peaks for apparent polyploid specimens were often of near to equal amplitudes and thus, as accurate allele determination is essential for population genetic analysis, all called alleles were kept for determining the genotype. This is problematic, as the allele dosage of polyploid samples cannot be assessed from the genotype of the sample. For instance, an individual sample appearing as genotype AB may indeed be AB if it is diploid, but AAB or ABB if it is triploid, and AAAB, AABB, or ABBB if tetraploid. As a result, we could not use standard inference of diploid genotypic data, but used Meirmans (2020) software Genodive version 3.05 to infer the ploidy level and dosage compensation of individual samples instead. Ploidy level was discerned by the maximum observed allele count per each sample by the software Genodive version 3.05 (Meirmans, 2020). Missing data were corrected by imputation and a maximum likelihood method was used to correct for the unknown dosage of the alleles using Genodive version 3.05 (Meirmans, 2020) implementing a modified version of the method of De Silva et al. (2005). As accurate estimation of allele frequencies of individuals and populations is the basis for most population genetic analyses, data based on dosage compensation were used here. Unless otherwise indicated, all analyses were conducted by using Genodive version 3.05 (Meirmans, 2020) and the significance of the estimated parameters was defined with permutation tests.

Clonal lineages were identified using a Stepwise Mutation Model with a threshold of 0 and set clones specific to every population. Tests for clonal population structure based on the concept of clonal diversity (Gómez & Carvalho, 2000) using Corrected Nei's diversity index were performed during clonal assignment. A total of 1228 multilocus genotypes (MLGs) were identified from the 1443 samples. Proportions of clones and shared clonal lineages were calculated manually. Clonal diversity was described as expected heterozygosity within populations (*H*_s_; Nei, 1987), the Shannon index (*shc*; both corrected for sample size [Chao & Shen, 2003]), the effective number of genotypes (*eff*) and the evenness of genotypes over the population (*eve*). Moreover, hidden clonal diversity was estimated with a rarefaction analysis using iNEXT Online (Chao et al., 2016) on the full dataset including clonal lineages (1000 permutations) and a confidence interval of 95%. Proportions of ploidy levels were calculated manually. To test the significance of ploidy levels across populations ploidy levels were defined as groups and the test statistic of *H*_s_ was used to compare between diploid, triploid and tetraploid groups (1000 permutations). All hereafter mentioned statistical tests were implemented on SPSS version 27.0.1.0 (IBM Corp, 2020). Two k independent‐samples Kruskal–Wallis tests were used to test the significance of ploidy levels across populations and loci. Pearson correlation was used to test the relationship between the number of alleles at a locus and the ability to detect polyploids. Two Mann–Whitney tests were used to test the differences in amounts of total MLGs and clonal MLGs between forms and a one‐sample t test was used to determine the difference in the number of clonal MLGs among clonal populations. Chi‐square crosstabulation was used to test the association between ploidy level (groups: diploid, polyploid) and clonality.

The following analyses were performed on two datasets, either including a single ramet per clonal lineage per population, or including all ramets, or on both datasets concurrently. Genetic diversity (*H*_s_) was estimated within populations and for the total population. Spatial genetic structure was assessed using several methods. First, spatial structure was described visually by conducting a principal component analysis (PCA), calculated from a covariance matrix (1000 permutations). Second, pairwise genetic differentiation between populations was estimated using *Rho*_ST_ index, which is analogous to *F*_ST_, but independent of the ploidy level (Meirmans, 2020; Ronfort et al., 1998). Significant differentiation (*Rho* > 0; 1000 permutations) was determined and manually corrected using Bonferroni correction. Third, isolation by distance (IBD) was determined by plotting pairwise genetic differentiation against pairwise geographic distances generated by the Geographic Distance Matrix Generator (Ersts, 2012). Significance of the matrix correlation was tested with Mantel's test (1000 permutations). IBD was assessed for the total data, and for both forms separately. Finally, we described the distribution of genetic variation with hierarchical Analysis of Molecular Variance (AMOVA; Excoffier et al., 1992). In the AMOVA, genetic variation was first allocated to different hierarchical levels and then the associated fixation indices of *Rho* and their significance (*Rho* > 0; 999 permutations) were determined. We used two alternative a priori hierarchies in AMOVA: (i) populations were nested within the form; (ii) populations were nested within subbasins. The latter analysis was performed separately for (A) attached and (B) free‐living populations.

## 3 RESULTS

### 3.1 Polyploidy

**FIGURE 2:**
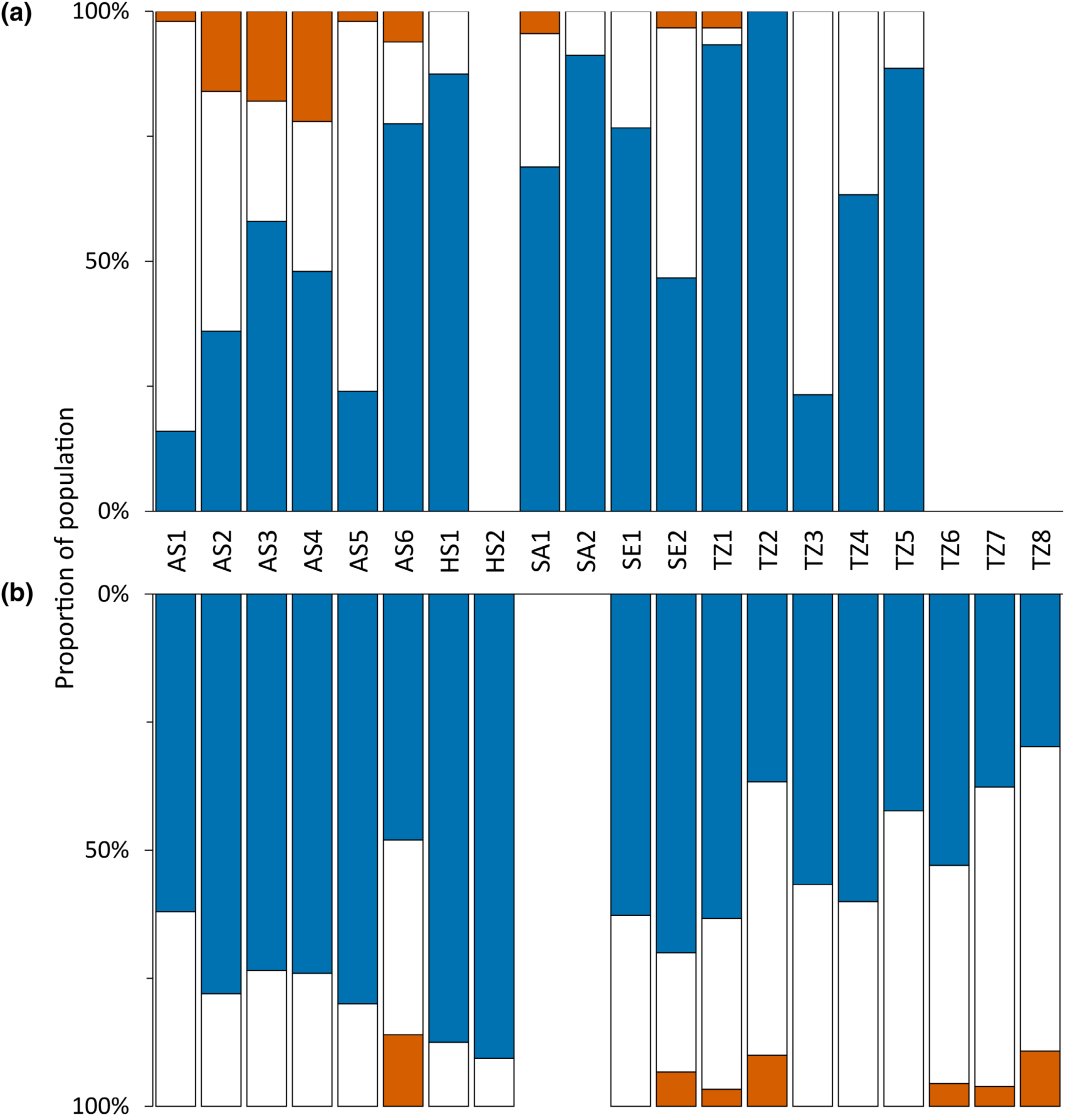
Frequency of ploidy levels in attached (a) and free‐living (b) *Fucus vesiculosus* populations [clones included]. Color representation: Blue, diploid; white, triploid; orange, tetraploid. AS, Askö; SE, Seili; TZ, Tvärminne; KU, Saaremaa; HS, Hiddensee.

We found potential signals of polyploidy in all populations except one (TZ2.F; Figure 2). The number of samples with ≥3 alleles differed significantly across populations (Kruskal–Wallis H 282.8, df 33, *p* < .001). The average frequency of potential polyploid samples was 38% (range among sites: 7%–84%) and over half of the samples appeared polyploid in five attached and six free‐living populations. The average frequency of potential polyploidy was similar in both forms (attached: 39%; free‐living: 38%). However, the frequency varied considerably both among subbasins and between forms at the same sampling site. On average, triploidy appeared more common than tetraploidy (triploid: 35%; tetraploid: 4%). Attached and free‐living populations had similar proportions of polyploidy (triploid, attached: 36%; free‐living: 34%; tetraploid, attached: 3%, free‐living: 5%). Genetic diversity (*H*_s_) did not vary significantly when individuals were grouped according to the defined ploidy level (diploid, triploid, tetraploid; Table 1).

**TABLE 1:** Comparison of genetic diversity (*H*_s_, heterozygosity within populations) between ploidy levels with *p* values generated by 1000 permutations.

	Diploid	Triploid	Tetraploid	*p* value
*H*_s_	0.710 (0.708)	0.708 (0.711)	4.429 (4.197)	.686 (.788)

*Note*: Values were calculated from the data where a single ramet per clonal lineage per population was included, values in brackets were calculated from the data where all ramets per clonal lineage per population was included.

Polymorphism of the loci affected the ability to detect potential polyploids (Appendix S4). There was a significant difference in the ability of each locus to detect ≥3 alleles (Kruskal–Wallis H 1179.928, df 7, *p* < .001, Appendix S4A) with greater allele variance at a given locus being weakly but non‐significantly associated with capturing ≥3 alleles (Pearson correlation .522, N 8, *p* .184; Appendix S4B). The determined ploidy level of the samples was significantly associated with the type of MLGs observed (Appendix S5). Diploid thalli were more likely to be associated with clonal MLGs than would be expected, while potential polyploids are more likely to be unique MLGs.

### 3.2 Clonality

**FIGURE 3:**
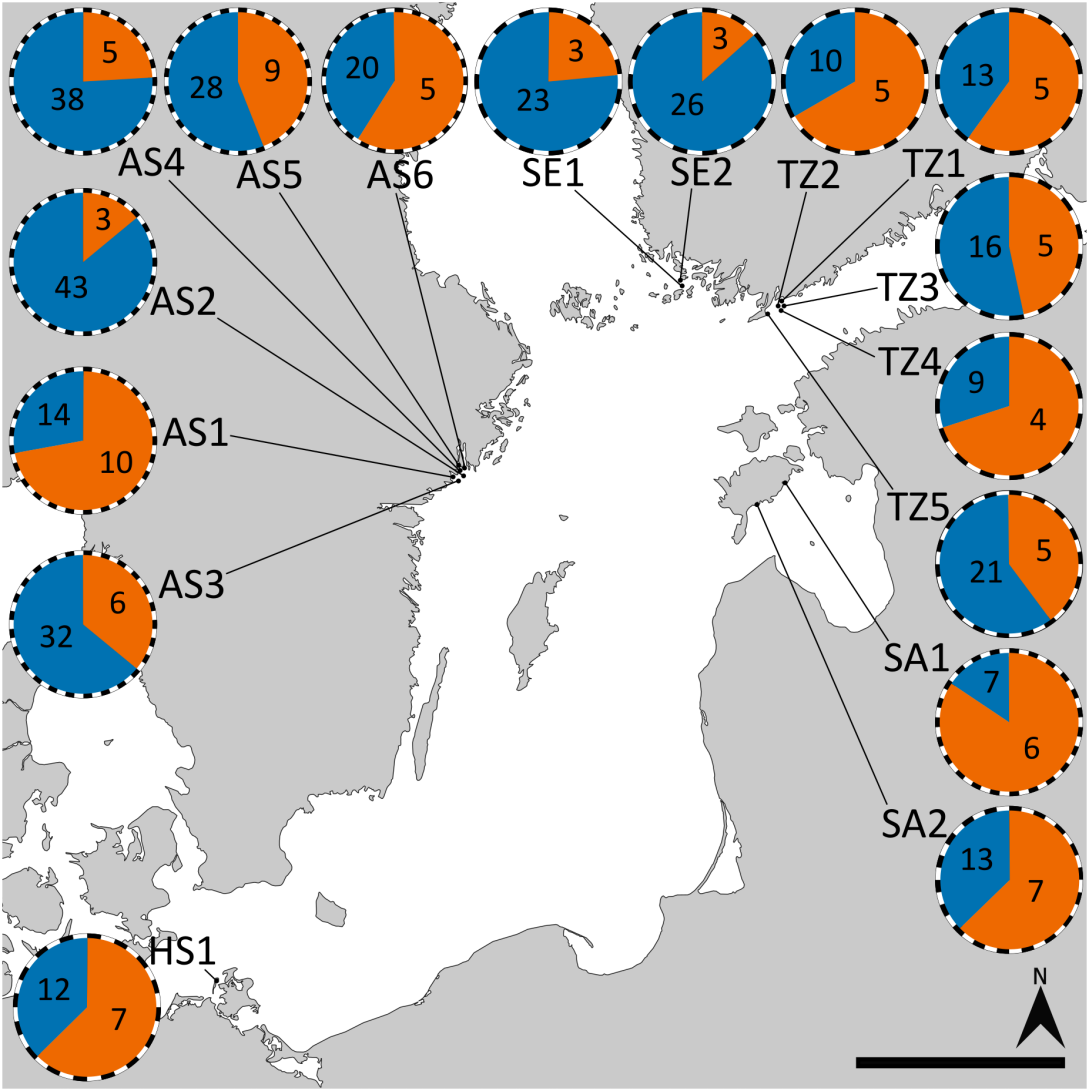
Proportion of clonal MLGs in the free‐living *Fucus vesiculosus* populations. Color representation: Blue, unique MLGs; orange, clonal MLGs. Dashed lines represent sample size at each site. AS, Askö; SE, Seili; TZ, Tvärminne; KU, Saaremaa; HS, Hiddensee. Numbers represent the amount of clonal or unique genotypes within free‐living populations.

**FIGURE 4:**
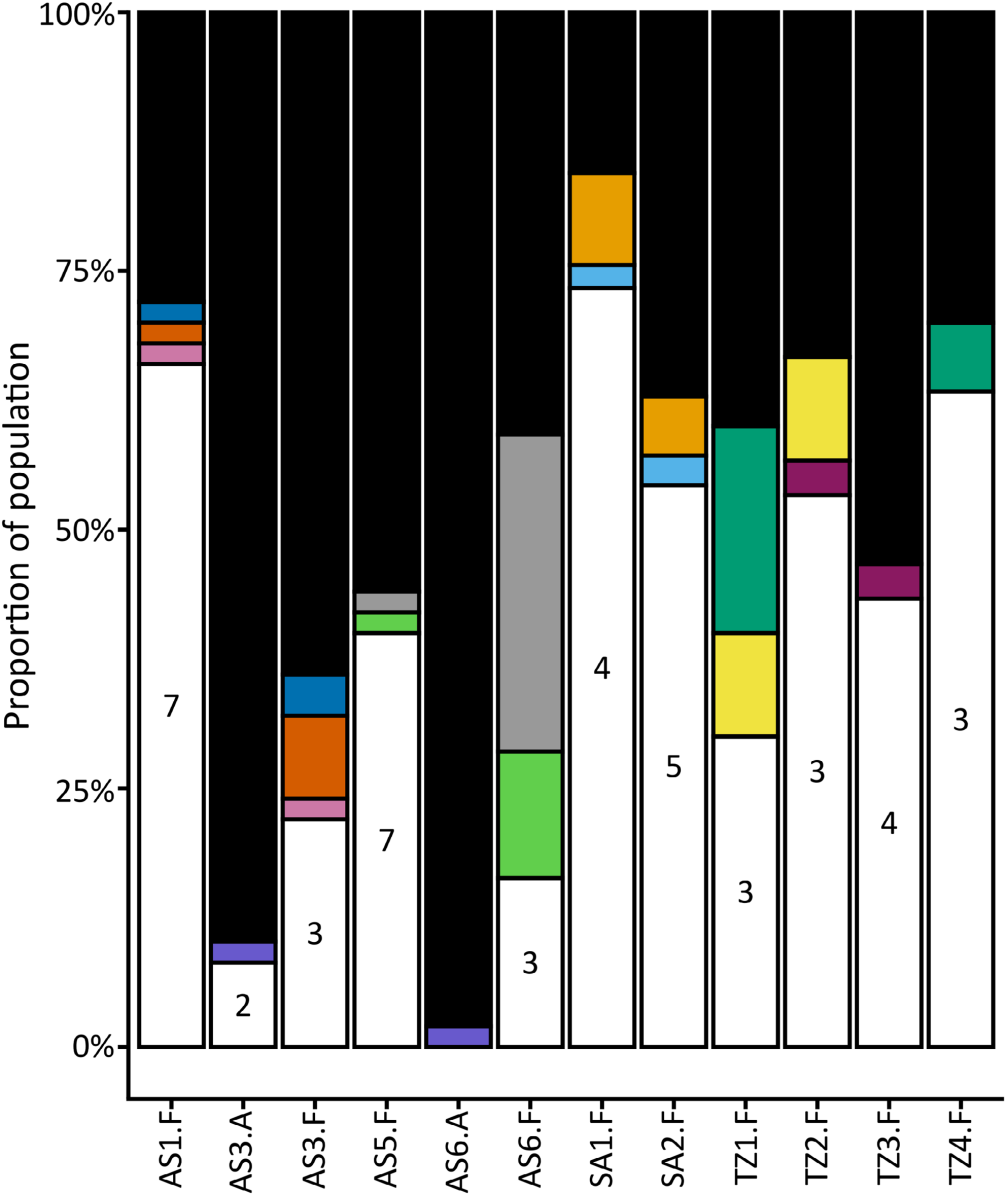
Proportions of shared clonal lineages in the *Fucus vesiculosus* study populations. Color representation: Black, unique MLGs; white, site‐specific clonal MLGs; colors, each represents a shared clonal MLGs found in multiple populations. Numbers in white portions indicate the number of site‐specific clonal genotypes represented. A, attached; F, free‐living; AS, Askö; HS, Hiddensee; SA, Saaremaa; SE, Seili; TZ, Tvärminne.

Clonal MLGs were found in three attached populations in Askö (AS3 [3], AS6 [1]) and Tvärminne (TZ8 [1]). These clonal MLGs represented 2%–10% of the total samples within each population and only 1% of the total attached sample. Tests of clonal diversity confirm that clonal MLGs at AS6 and TZ8 are likely per chance identical genotypes as a result of random mating while clonal MLGs at AS3 likely represent true clonal lineages (Appendix S6). The two populations at Askö (AS3, AS6) shared a single clonal MLG. All free‐living populations were multiclonal, but in contrast to attached populations, almost half of the samples belonged to site‐specific or shared clonal MLGs. The frequency of clonal MLGs in free‐living populations varied significantly across populations (*t* = 9.061, df = 18, *p* < .001; range: 13%–84%, Figure 3). Single clonal MLGs were rarely dominant, even in populations with high clonality. The majority of clonal MLGs were rare (Appendix S7; mean ramet number per genet = 4) although the most abundant two clonal MLGs represented 52% [AS6] and 45% [SA1] of the site‐specific clonal population. However the representation of clonal MLGs varied strongly across populations (e.g. six clonal MLGs represented 36% (AS3) to 84% (SA1) of the total population; Figure 4). Some clonal MLGs were shared between populations from the same subbasin (Tvärminne, Saaremaa, Askö), but never across subbasins (Figure 4). Of the free‐living populations 10 shared clonal MLGs. Patterns of shared clonal MLGs were similar in these free‐living populations, with each population sharing 1–2 clonal lineages. Shared clonal MLGs were not widespread, being only observed in a maximum of two populations. Clonal diversity, represented by the genetic diversity indices (*H*_s_ and *shc*), was high in all free‐living populations (Table 2). Testing the probability of finding the observed clonal diversity under random mating shows that all free‐living populations except for TZ1 deviate from what would be expected under random mating (Appendix S6), suggesting that they are true clones. Clonal MLGs were not shared among the two forms.

**TABLE 2:** Clonal diversity statistics for the free‐living populations

Population	Num	Eff	Eve	*H*_s_	Shc
AS1_F	25	12.500	0.500	0.939	1.454
AS2_F	44	36.765	0.836	0.993	2.317
AS3_F	39	29.070	0.745	0.985	2.004
AS4_F	44	36.765	0.836	0.993	2.317
AS5_F	39	29.070	0.745	0.985	2.001
AS6_F	25	7.872	0.315	0.891	1.433
HS1_F	19	12.190	0.642	0.948	1.442
SA1_F	13	4.698	0.361	0.805	0.979
SA2_F	19	11.036	0.581	0.936	1.385
SE1_F	26	22.500	0.865	0.989	2.032
SE2_F	28	26.471	0.945	0.995	2.328
TZ1_F	19	12.500	0.658	0.952	1.478
TZ2_F	14	5.422	0.387	0.844	1.165
TZ3_F	20	12.857	0.643	0.954	1.540
TZ4_F	14	6.250	0.446	0.869	1.173
TZ5_F	27	19.444	0.720	0.976	1.833

Abbreviations: AS, Askö; *Num*, number of genotypes; *eff*, effective number of genotypes; *eve*, evenness; *H*_s_, heterozygosity within populations; *shc*, Shannon index corrected for size; SE, Seili; TZ, Tvärminne; KU, Saaremaa; HS, Hiddensee.

The proportion of MLGs detected was significantly larger in the attached form (Appendix S8A). Rarefaction analysis shows that the presence of clones within the free‐living form influenced the ability to capture the genetic diversity in the populations (Appendix S9). To capture a similar genotypic diversity in the free‐living populations as was captured for the attached populations, the sampling effort in this study should have been greater than the extrapolated maximum, at least doubling that of the current sampling effort.

### 3.3 Intraspecific variation

Genetic diversity across the subbasins (*H*_s_) did not differ significantly (range: 0.58–0.64), but *H*_s_ differed significantly between the two forms (Table 3). Genetic diversity was greater in the attached population compared to the free‐living one, and the difference between the forms was boosted when all ramets of the clonal MLGs were included in the analysis.

**TABLE 3:** Genetic diversity within groups (total population, by subbasin, by form), *p* values determine the statistical significance of variation within the grouped values.

	*H*_s_
Whole population	0.613 (0.601)
Askö	0.647 (0.634)
Hiddensee	0.617 (0.600)
Saaremaa	0.623 (0.573)
Seili	0.636 (0.637)
Tvärminne	0.576 (0.566)
*p* value	.611 (.540)
Attached	0.643 (0.643)
Free‐living	0.582 (0.555)
*p* value	.008 (.001)

*Note*: Maximum likelihood method was used to correct for the unknown dosage of the alleles. Values were calculated from the data where a single ramet per clonal lineage per population was included, values in brackets were calculated from the data where all ramets per clonal lineage per population were included. Abbreviations: *H*_s_, heterozygosity within populations.

**FIGURE 5:**
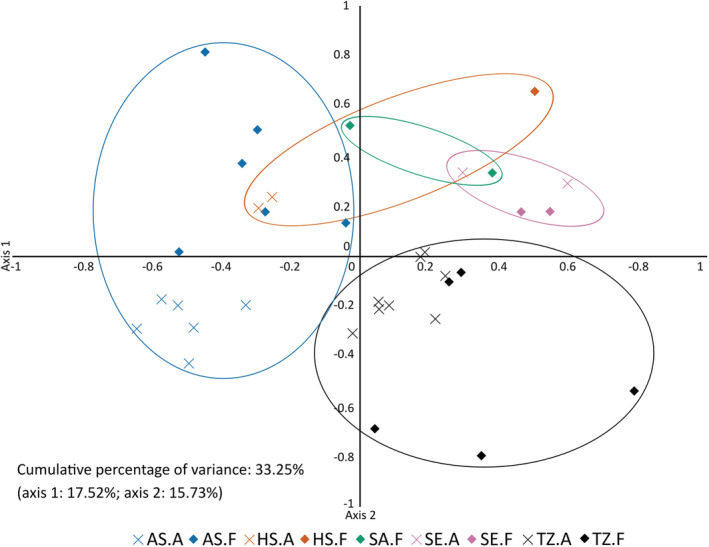
PCA based on allele frequencies within *Fucus vesiculosus* populations. First and second axis plotted. A, attached; F, free‐living; AS, Askö; HS, Hiddensee; SA, Saaremaa; SE, Seili; TZ, Tvärminne.

In the PCA, the first two principal components explained 33% of the variance in the data. Populations from subbasins Tvärminne, Askö and Seili were loosely clustered with each other. Askö populations were separated from the Finnish populations on the first principal component and Tvärminne and Seili populations from each other on the second principal component (Figure 5). Within each subbasin, the forms generally also grouped more closely together. The rest of the subbasins did not form consistent clusters.

**FIGURE 6:**
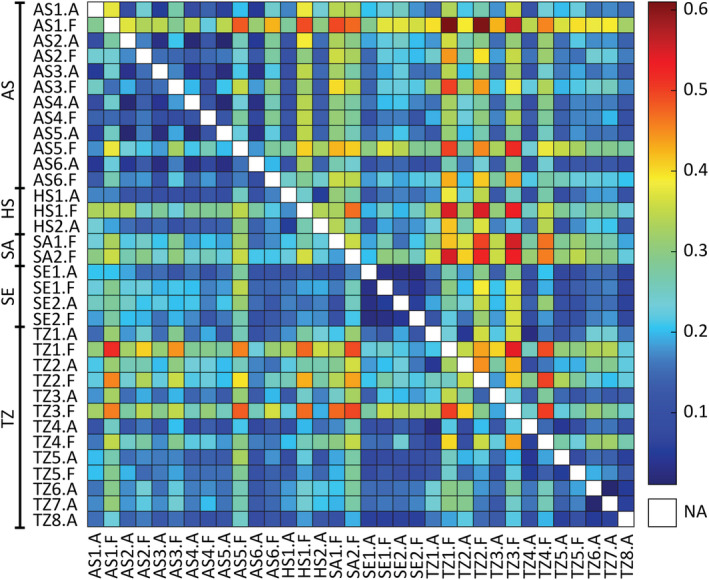
Pairwise *Rho*_ST_ genetic distance matrix between 34 *Fucus vesiculosus* populations. Abbreviations: A, attached; F, free‐living; AS, Askö; HS, Hiddensee; SA, Saaremaa; SE, Seili; TZ, Tvärminne. Numbers represent sympatric sites within a subbasin. *p* values all >.05 with Bonferroni correction.

**FIGURE 7:**
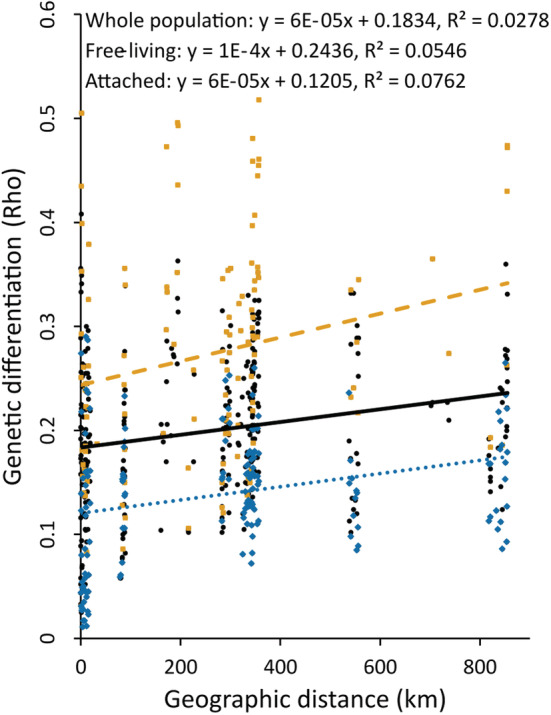
Isolation‐by‐distance in the whole *Fucus vesiculosus* population (black circle; solid line), and separately in free‐living (orange square; dashed line) and attached (blue rhombus; dotted line) populations. Pairwise genetic differentiation (*Rho*_ST_, X axis) plotted against geographic distances (km, Y axis).

Pairwise genetic distances (*Rho*_ST_) varied widely, with a mean *Rho*_ST_ of 0.20 including a single ramet per clonal lineage per population (range: 0.01–0.52) and 0.24 including all ramets (range: 0.02–0.61; Figure 6). Within subbasins, there was a general trend of greater differentiation between populations from different forms compared to the same form, except for Seili, where differentiation was low irrespective of form. Differentiation among closely located attached populations was low, particularly in Askö, while differentiation among attached populations from different subbasins was greater. Free‐living populations show a less uniform pattern of differentiation, but the largest pairwise *Rho*_ST_ values came from comparisons between free‐living populations, both within and among subbasins. A significant IBD signal was found in the attached form (*p* .010) and in the whole data (*p* .007), but not in the free‐living form (*p* .119, Figure 7).

In AMOVA, there was significant spatial structuring at all hierarchical levels, both between forms and among each form (Tables 4 and 5). However, the forms were strikingly different in their pattern of spatial structuring. Differences between populations within the forms explained much more of the total variance than the differences between the forms themselves. Likewise, genetic differentiation (*Rho*_ST_) was an order of magnitude larger within compared to between forms (Table 4). Moreover, forms also showed a distinctly different pattern when populations were nested within subbasins and forms were analyzed separately. Both forms had similar genetic differentiation and a similar amount of variation allocated to the among subbasins level. However, differentiation and the amount of variation increased about three fold at the within‐subbasin level in the free‐living form, while remaining the same in the attached form (Table 5).

**TABLE 4:** Hierarchical AMOVA (I) when populations were nested within forms

	Variance explained by	df	% of variance	*F*‐index	*p* value
*Rho*_ST_	Within population	1194	0.815	0.185	–
*Rho*_SC_	Among population nested within form	32	0.173	0.175	.001
*Rho*_CT_	Among forms	1	0.013	0.013	.001

Abbreviation: df, degrees of freedom.

**TABLE 5:** Hierarchical AMOVA (II) when populations were nested within subbasin, separately for attached (a) and free‐living (b) populations

		A: Attached				B: Free‐living			
*F*‐statistic	Variance explained by	df	% of variance	*F*‐value	*p* value	df	% of variance	*F*‐value	*p* value
*Rho*_ST_	Within population	793	0.840	0.160	–	399	0.722	0.278	–
*Rho*_SC_	Among population nested within subbasin	14	0.073	0.080	.001	11	0.196	0.214	.001
*Rho*_CT_	Among subbasins	3	0.087	0.087	.001	4	0.082	0.082	.001

Abbreviation: df, degrees of freedom.

## 4 DISCUSSION

In this study, we demonstrate that two forms of Baltic Sea *F. vesiculosus* are markedly different in their genetic characteristics. Overall, genetic diversity was similar throughout our study area, albeit significantly different between forms. Clonality was virtually absent from the attached form, while being far more common in the free‐living populations, which also shared clonal lineages within subbasins. Contrary to our expectation, signatures of polyploidy appeared evident throughout the majority of populations, irrelevant of form. The overall population was structured by both form and subbasin, but the free‐living populations were more strongly spatially structured within subbasins compared to the attached populations. While corroborating that the forms belong to the same species, their varying genetic characteristics suggest that their reproductive modes, dispersal capabilities, and population connectivity differ. Thus, our results highlight the necessity to manage the genetic diversity of each form with independent but congruous efforts.

### 4.1 Genetic diversity in the Baltic Sea *F. vesiculosus*

When using predominantly the same DNA microsatellite markers, our results showed that the genetic diversity was at the same level (*H*_s_ 0.44–0.69) when compared to previous studies in the Baltic Sea (Johannesson et al., 2011; Pereyra et al., 2013; Tatarenkov et al., 2007) and globally (Perrin et al., 2007; Wallace et al., 2004). As expected based on the assumed dominance of asexual reproduction in the free‐living form (Bauch, 1954; Svedelius, 1901), the free‐living populations were significantly less genetically diverse than their attached counterparts. Nevertheless, genetic diversity was still within expected limits for the species in both forms. Thus, frequent asexual reproduction in the free‐living form did not drastically reduce the overall genetic variation in *F. vesiculosus*.

### 4.2 Polyploidy in the Baltic Sea *F. vesiculosus*

Polyploidy has not been found in previous genetic studies of Baltic Sea *F. vesiculosus* (Ardehed et al., 2016; Johannesson et al., 2011; Pereyra et al., 2009; Rinne et al., 2018; Tatarenkov et al., 2005, 2007), but has infrequently been reported elsewhere in *Fucus* spp. (Coyer, Hoarau, Pearson, et al., 2006; Gómez Garreta et al., 2010; Sjøtun et al., 2017). Based on these findings, we did not expect to find variation in the ploidy level in our study, yet a considerable portion of our samples were determined to possess ≥3 alleles in at least one locus. We verified this finding by meticulously scrutinizing the multiple‐peak patterns in the electropherograms and by reanalysing samples, showing that these patterns were repeatable and consistent.

In natural populations, tetraploidy is generally the most common polyploid level (Comai, 2005), but instead, we observed only a few tetraploids (4%) and extensive triploidy (35%). It must be noted, however, that our assessment of the ploidy level is based on using genetic markers and higher ploidy levels can be missed because allele dosage cannot always be discerned. For example, a heterozygote phenotype AB in the electropherogram may be genotype AB if diploid, AAB or ABB if triploid, and AAAB, AABB, or ABBB if tetraploid. Similarly, a single peak in the electropherogram may denote genotype A if haploid, AA if diploid, AAA if triploid and AAAA if tetraploid. Thus, the level of ploidy is probably downgraded among our samples and particularly the low proportion of tetraploids may not be accurately estimated. As our a priori assumption of *F. vesiculosus* being diploid in the Baltic Sea appeared incorrect, we could only employ a posteriori methods designed to accommodate polyploid data in mixed‐ploidy populations, i.e. use dosage compensation (Meirmans, 2020). This emphasizes the need to assess the level of ploidy in the samples when polyploidy is suspected, using e.g. micro spectrofluorometry or flow cytometry. Further study is warranted to affirm these signatures of polyploidy within Baltic Sea *F. vesiculosus*.

Polyploidy can arise as hybridization between two (or more) related species (allopolyploidy), or it can be a result of multiplication of the whole genome of a single parent species (autopolyploidy). In the Baltic Sea, *F. vesiculosus*, *F. radicans* and *F. serratus* are native, and *F. evanescens* is an invasive species (Bergström et al., 2005; Malm et al., 2001; Wikström et al., 2002), which suggests that both allopolyploid and autopolyploid forms can potentially arise. Outside the Baltic Sea, *Fucus* species are typically separated by intertidal zonation (Colman, 1933; Fritsch, 1945; Lubchenco, 1980; Zaneveld, 1937) and species occur at the same geographical sites, whereas in the Baltic Sea, the distribution of different *Fucus* species is increasingly controlled by the salinity gradient (Isæus, 2004). This means that large sections of the Baltic coastal zone are dominated by a single species and sympatry is uncommon outside the tolerance margins of each species.

In our study area, the Arkona Basin (Germany, Hiddensee) and the Gulf of Riga (Estonia, Saaremaa) are the only known areas with a potential for multiple species occurring sympatrically. Apart from *F. vesiculosus*, *F. evanescens* (Dietrich and Schubert, 2017; Lackschewitz et al., 2013; Schueller and Peters, 1994) and *F. serratus* (HELCOM, 2013) have been reported in the Arkona Basin, although later surveys have failed to find them (Dietrich and Schubert, 2017; HELCOM, 2013). Thus, it is unlikely that *F. evanescens* or *F. serratus* form sympatric populations with *F. vesiculosus* along the German Baltic coast. Both species are also genetically distinct from *F. vesiculosus* (Coyer, Hoarau, Oudot‐Le Secq, Stam, Olsen, 2006), and hybridization between these species and *F. vesiculosus* would result in genetic differentiation of the Hiddensee populations from all other sites. As this was not the case, hybridisation between multiple species as a source of polyploidy seems unlikely in the Hiddensee population.

In the Gulf of Riga, *F. vesiculosus* and *F. radicans* occur both sympatrically and allopatrically (Johannesson et al., 2011; Pereyra et al., 2013), and both allopolyploidy and autopolyploidy are possible origins of polyploidy in this area. There are several pieces of evidence that favor autopolyploidy in the Gulf of Riga populations. First, other *Fucus* spp. populations are geographically sufficiently separated from our Gulf of Riga sites; second, as above, genetic differentiation between the Gulf of Riga populations and our other study sites was relatively low; third, the occurrence of polyploidy was analogous to the rest of the populations. Thus, autopolyploidy is a more likely origin of polyploidy also in the Gulf of Riga populations, but further research is needed to confirm this.

Frequent fusion of reduced and unreduced gametes (Bretagnolle and Thompson, 1995) alongside the advantages of polyploidy (heterosis, gene redundancy, asexual reproduction; Comai, 2005) may be supporting the apparent triploidy. Several natural plant populations are known to be dominated by triploids (Kim et al., 2016; Lee et al., 2016; Mock et al., 2012) and thus the high amounts of observed triploids within our populations are not wholly unexpected. As triploids are unstable and frequently sterile (Ramsey and Schemske, 1998), the many unique MLGs in the attached populations would suggest that the benefits of polyploidy are not large enough to allow for triploids to dominate attached populations. Conflicting this, the high frequency of triploidy in free‐living populations appears more logical, because infertile triploids can propagate vegetatively (Pearson, 2001). After a single event of triploid zygote formation, several events of asexual reproduction by the mature triploid thallus may lead to multiple triploid clones within the free‐living population. As triploids are often more vigorous with higher fitness compared to diploids (Miller et al., 2012), triploid clones could spread rapidly through the population. However, if polyploids receive a fitness boost, it could be argued that tetraploids should be more abundant as they are both stable and fertile (Comai, 2005), this should apply to both sexually and asexually reproducing populations. Although within algae, increasing the genome size may not to provide a fitness advantage. Polyploid *Gracilaria tikvahiae* have lower fitness than their diploid conspecifics and increasing genome size (triploidy vs. tetraploidy) results in an even greater reduction of fitness (Patwary and van der Meer, 1984; van der Meer and Patwary, 1983; Zhang and van der Meer, 1988). Thus, the rarity of tetraploidy compared to triploidy may be valid or an artifact resulting from the resolution of the genetic markers used.

### 4.3 The occurrence of clonality in *F. vesiculosus*

Many of our study populations included samples that shared their multilocus genotype. As the probability of clonal MLGs arising under random mating within most populations was minimal (Appendix S6), samples sharing genotypes can be considered true clones (ramets). Thus we describe them as clonal MLGs. As algae are known to asexually reproduce through various methods (e.g. stoloniferous growth, fragmentation, adventitious branches [Collado‐Vides, 2001; Fritsch, 1935, 1945]) the exact mechanisms underlining clonal production cannot be determined from microsatellite analysis alone. We suggest that the fragility of *F. vesiculosus* thalli, particularly of the free‐living form (R. Preston, pers. comm.), alongside the frequent occurrence of adventitious branches (Kinnby et al., 2019) indicates that fragmentation and/or adventitious branches are credible methods. As the free‐living form has previously been presumed to be sterile (Bauch, 1954; Häyrén, 1949; Svedelius, 1901), the origin of clonal MLGs would therefore have to be either entirely or predominantly through clonal growth.

The proportion of clonal MLGs significantly varied among populations and was far larger in free‐living populations compared to attached populations on average (Appendix S8B). In attached populations, we found clonal MLGs only in three populations and the average amount was small (1%). This is less than in previous studies on attached Baltic Sea *F. vesiculosus*, where most populations studied had clonal MLGs and the overall proportion was larger (7%–36%; Ardehed et al., 2016; Johannesson et al., 2011; Tatarenkov et al., 2005). In fact, the proportion of clones in the studies above resembles more what we found in the free‐living populations, where clonal MLGs were found in all populations and some were dominated by clonal MLGs. A number of clonal MLGs were found in more than one population. These were always located within the same subbasin, i.e. within a restricted geographic region, but clonal MLGs were not shared with pairs of attached and free‐living populations. Previously, only Johannesson et al. (2011) reported shared clonal MLGs between closely located *F. vesiculosus* populations. The presence of shared clonal MLGs are a direct indication of dispersal between populations and will be discussed below.

Asexual reproduction has been suggested to increase towards the range margins of the species (Billingham et al., 2003; Eckert, 2001; Kearney, 2003). In the studies above, many of the study populations were located north of our study area, in the Bothnian Sea, and the lower salinity of these study sites may have contributed to the higher prevalence of clonal MLGs. However, one of our attached populations with a higher amount of clonal MLGs (AS3) was not among the sites with lowest salinity. Additionally in attached populations at Öland and Öregrund, with similar salinity ranges to our study sites, clonal MLGs were pervasive (Ardehed et al., 2016; Johannesson et al., 2011; Tatarenkov et al., 2005). Interestingly, both Ardehed et al. (2016) and our study found that clonality was absent for the attached form in the Archipelago Sea. Thus, it is possible that the prevalence of clonality varies due to selective and/or neutral processes (Rafajlović et al., 2017) in attached populations in the Baltic Sea and is rare at our study sites.

### 4.4 Spatial genetic structure

The Baltic Sea *F. vesiculosus* population was structured at various levels, with both spatial factors and form influencing the connectivity among populations. When the data were organized according to the forms (populations nested within forms), forms were significantly genetically differentiated from each other. However, this hierarchical level explained only a minor part (1%) of the total variance in the data and the among‐populations level explained more than an order of magnitude more (17%, Table 4). When attached and free‐living populations were analyzed separately in the AMOVA, populations were significantly differentiated both within and among the subbasins in both forms, but the spatial structuring among local populations within the subbasins was clearly stronger in the free‐living compared to the attached form. Moreover, the attached populations showed a weak but significant isolation‐by‐distance effect, as expected when gene flow does not cover the whole study area.

Previous results on spatial genetic structuring of the Baltic Sea *F. vesiculosus* population have been somewhat mixed. Our work is in line with previous results showing strong structure among spatially closely located populations along the Swedish east coast (Pereyra et al., 2009; Tatarenkov et al., 2007), the Finnish west coast (Rinne et al., 2018), and also at larger scales (Ardehed et al., 2016; Johannesson et al., 2011). Contrary to this, a wide‐scale study only found significant genetic structuring among local attached *F. vesiculosus* populations in the Gulfs of Bothnia and Riga, but not among the regions (Pereyra et al., 2013). Previous results on the effects of geographic components on the spatial genetic structure have also been mixed. Tatarenkov et al. (2007) showed an isolation‐by‐distance signal at both local (<10 km) and large (<1000 km) scales in *F. vesiculosus*, while (Ardehed et al., 2016) did not find such signal in *F. vesiculosus* nor *F. radicans*, although the scale and distribution of these studies were not directly comparable.

Our results on the spatial genetic structuring suggest that the gene flow between the two *F. vesiculosus* forms is not completely free with the two forms representing different spatial genetic structures. The spatial genetic structure within the free‐living form appears to be increasingly driven by the strong genetic differentiation among local free‐living populations, while gene flow is equally restricted at both large and small spatial scales in the attached form. Free‐living populations thus appear far more isolated at a local scale indicating geographically close populations may have markedly different origins with genetic differences maintained by clonal growth. Our results also suggest a difference in gene flow between the two forms. In the attached form, gene flow extends further than in the free‐living form and it seems that it is not restricted to the scale of subbasins, which shows as a significant IBD signal. On the contrary, gene flow in the free‐living form appears to be random and more restricted to the within subbasins scale. This effect can be partly explained by the fashion in which these populations emerge; if new free‐living populations are founded by a small number of individuals, genetic differentiation among populations increases due to the founder effect. Another possible explanation for this difference is the potential for dispersal in the forms. Attached populations predominantly reproduce sexually, which means that most dispersal takes place by actively released sexual propagules, which attach to the bottom in the target population. Conversely, sexual reproduction would seem to be limited in the free‐living form and dispersal takes place largely by freely floating pieces of detached thalli. A difference in the dispersal of sexual propagules and detached thalli would thus contribute to the observed difference in the gene flow. The origin of free‐living populations is discussed next in more detail.

### 4.5 Origin and maintenance of free‐living *F. vesiculosus*

It is conceivable that free‐living *F. vesiculosus* populations emerge either by asexual (clonal growth) or sexual reproduction and subsequent dispersal of propagules to a new location. Both modes of reproduction could take place in either attached or free‐living populations and moreover, these alternatives are not mutually exclusive. Our study does not provide direct answers to the origin of the free‐living populations, but clonal diversity and distribution, and the spatial structure of the population allow us to discuss these scenarios. Free‐living forms of *Fucus* spp. populations have classically been assumed to derive from attached populations through clonal growth whereby pieces of thalli (either as pieces of typical adult thalli or adventitious branches) would detach from attached individuals, float freely and eventually aggregate in still locations and persist over a longer time (Bauch, 1954; Cotton, 1912; Den Hartog, 1959; Fritsch, 1945; Häyrén, 1949; Luther, 1981; Svedelius, 1901). Alternatively, pieces of thalli may detach from other free‐living populations and eventually aggregate in a new location.

Another scenario for the origin of free‐living populations involves sexual reproduction in attached populations and, theoretically, also in free‐living populations. Instead of attaching to the substratum, zygotes could attach to an inadequate anchoring surface leading to subsequent detachment (Baker and Bohling, 1916; Boney, 1966; Chapman and Chapman, 1973; Fritsch, 1945) or they may settle in a quiet environment where attachment substrates are unavailable and develop in situ (Lee, 1989). The typically soft sediment dominated coastal environments associated with free‐living populations provide unfavorable conditions for colonization from small sexually recruited stages due to the effects of sedimentation including burial, altered light conditions and altered chemical micro‐environment (Berger et al., 2003; Chapman and Fletcher, 2002; D'Antonio, 1986; Daly and Mathieson, 1977; Devinny and Volse, 1978; Eriksson and Johansson, 2003). In fact, recruitment of new *F. vesiculosus* in soft sediment dominated environments has previously been unsuccessful (Shaughnessy, 1982). Unique MLGs found in free‐living populations could be contributed by sexual reproduction of free‐living thalli, but there is no direct evidence of sexual reproduction in free‐living populations. The closest available comparison comes from an embedded *Fucus* population in the western Baltic Sea near Glücksburg, which is largely infertile in the wild and maintains low fertility under laboratory conditions (Meichssner et al., 2021). Thus, free‐living populations most likely emerge from detached pieces of thalli aggregating in sheltered locations, but further tests are required to confirm zygote viability and successful development of unattached zygotes into mature free‐living individuals on suboptimal substrates.

There are indications that unattached algal populations can partially maintain themselves once a small input of source material has aggregated (Lobban and Harrison, 1997). Free‐living *F. vesiculosus* populations could be maintained with the same manner they originally arose, through clonal growth of resident free‐living thalli or by a continued supply of detached pieces of thalli from other populations. In fact, clonal growth may be the only option for free‐living *F. vesiculosus* when the soft sediment prevents attachment and hinder the survival of small sexual stages. If free‐living populations were solely maintained by within population clonal growth, they would be expected to be locally differentiated (Neiva et al., 2012) and dominated by one to a few clones as a signature of recurrent asexual reproduction (Barrett, 2015). Several free‐living populations are locally differentiated from both nearby attached and free‐living populations. However the majority of free‐living populations constitute a mosaic of mostly poorly represented clonal MLGs and a myriad of unique MLGs. This indicates the founder effect and passive clonal growth resulting from random proliferation of many resident genets. Clonal growth is generally an effective mode of reproduction and allows rapid colonization and domination of habitat patches. It poses clear short‐term fitness advantages, but is potentially inferior in the long run due to the limited possibilities to adapt to changing environments. As the longevity of free‐living populations is poorly understood, the consequences of recurrent asexual reproduction on the population is questionable.

If free‐living populations are supplemented by other populations, they are expected to share clonal MLG's with other populations and consequently, spatial genetic structure is expected to be shallow. Our results were almost completely the opposite, however. The proportion of clonal MLGs shared by multiple populations was low and populations were significantly differentiated from each other, which suggests no extensive supply from neighboring attached populations to maintain free‐living populations. However, finding many unique MLGs in free‐living populations is contrary to this, because vegetative growth is expected to rule out unique MLGs (see above). In one potential scenario, propagules with different genotypes gather in sheltered locations and free‐living population arise when emerging individuals fragment further at their own pace, resulting in a mix of clonal and unique MLGs. In an alternative scenario, detached pieces of thalli from one or more populations immigrate to a free‐living population. This would result in unique MLGs in the free‐living population, but shared clonal MLGs would then also be expected. While we confirmed that the nearest attached populations were not sources of the free‐living populations due to the lack of shared clonal MLGs, this scenario is feasible, if the immigrants are derived from a larger area and founder effect boosts genetic differentiation among populations. Detached thalli have a large dispersal capability (Rothäusler et al., 2015, 2020), but this scenario could be verified only by extending the study area considerably. It seems that once a free‐living population has emerged, it is maintained in multiple ways. Clonal growth is recurrent within the free‐living populations, but additional supply from other populations must also be relatively frequent. Overall it seems that free‐living populations are heterogeneous and processes maintaining the populations vary.

To conclude, attached populations predominantly reproduce sexually, and connectivity among populations is often higher than in the free‐living form. This suggests that the attached populations are able to maintain genetic diversity and adaptive potential in the face of changing environmental conditions as a network of populations. Conversely, free‐living populations are often dominated by clonal lineages with many populations showing greater isolation, resulting in decreased genetic diversity. Nevertheless, clonally dominated free‐living populations may benefit temporarily from the predicted changes in the salinity regimes and increasing temperature within the Baltic Sea (Meier et al., 2021). If one genotype is particularly well‐suited to the new conditions, asexual reproduction would allow rapid colonization by this genotype (Lasker and Coffroth, 1999; Wulff, 1991). In the long run, it is more likely that populations deprived of genetic diversity are vulnerable, as clonally reproducing organisms often are less able to adapt to changing environments (Nieuwenhuis and James, 2016). Free‐living populations are more unstable than their attached counterparts because the free‐living form is generally found in sheltered, shallow areas, often close to shore, which are particularly vulnerable to the effects of environmental change and eutrophication (Brito et al., 2012). Whole populations have also been lost in unfavorable flood situations (Bauch, 1954) or winter storms (Norberg, 1995), which have also been predicted to increase under climate change (Meehl et al., 2007). Consequently, free‐living populations show an increased vulnerability to local extinctions compared to the attached populations, emphasizing the need to consider the form independently.

## AUTHOR CONTRIBUTIONS

**Roxana Preston:** Conceptualization (equal); data curation (lead); formal analysis (lead); funding acquisition (lead); investigation (lead); methodology (lead); project administration (lead); resources (equal); supervision (equal); visualization (lead); writing – original draft (lead). **Jaanika Blomster:** Conceptualization (equal); supervision (equal); writing – review and editing (equal). **Ellen Schagerström:** Conceptualization (equal); resources (equal); writing – review and editing (equal). **Perttu Seppä:** Formal analysis (supporting); writing – review and editing (equal).

## FUNDING INFORMATION

Funding for this project was provided through grants from the Walter and Andrée de Nottbeck Foundation and the Onni Talas Foundation.

## CONFLICT OF INTEREST

The authors declare that they have no known competing financial interests or personal relationships that could have appeared to influence the work reported in this paper.

### OPEN RESEARCH BADGES

This article has earned an Open Data badge for making publicly available the digitally‐shareable data necessary to reproduce the reported results. The data is available at https://doi.org/10.6084/m9.figshare.19361759.

## Supporting information


Appendix S1

## Data Availability

Individual genotype data are openly available on Figshare: https://doi.org/10.6084/M9.FIGSHARE.19361759.
