# Supplementary material for: Clonality, polyploidy and spatial population structure in Baltic Sea Fucus vesiculosus
Source: Ecol Evol. 2022 Sep 20;12(9):e9336. doi: 10.1002/ece3.9336 (PMC9486819; doi:10.1002/ece3.9336)
Supplement: Supplementary file 1 — Appendix S1 [file ECE3-12-e9336-s001.docx]

**Appendix**

| y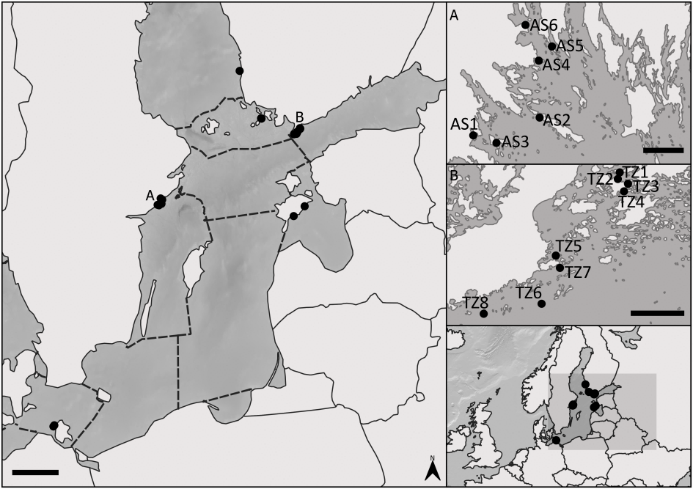 |
| --- |
| S1: Locations of the 20 sampling sites within the Baltic Sea. Black dots represent each sampling site. Scale bar: 100 km. Inset A represents the six sampling locations at Askö, inset B represents the 11 sampling locations at Tvärminne, scale bar: 5 km. Dashed lines indicate divisions of subbasins. |

**Full genotyping protocol**

Forward primers were fluorescently tagged at the 3’ or 5’ end (S3). PCR amplifications were split into single or multiplex reactions (S3). All primers were diluted to 10µM. Amplification was performed in 12.5µl reaction mixes, with each reaction containing 6.25µl polymerase master mix, 0.5ul of fluorescently tagged forward primer and 0.5µl reverse primer for each primer pair, 2.5µl bovine serum albumin (0.2mg/ml) and MQH_2_O to make up to 12µl.Multiplex reactions containing L85 and L94 differed with 0.6µl of forward and reverse L85 primer and 0.4µl forward and reverse L94 primer added to each reaction. For all reactions DNA was diluted 1:10 in MQH2O to a final concentration between 0.1-55.1ng/µl and 0.5µl of this dilution was directly added to each reaction well. Two different polymerases were used: OneTaq® 2X Master Mix with Standard Buffer (New England Biolabs, M0482L) or OneTaq® Hot Start 2X Master Mix with Standard Buffer (New England Biolabs, M0484L) (S3). All PCR reactions shared a common initial denaturation step of 95^o^C for 5min and final extension step of 72^o^C for 5min. PCR conditions were specifically tailored for the different primers pairs (S3). Programs were run either on a Veriti 96-Well (Applied Biosystems) or S1000 (Bio-Rad) thermal cycler. Failed multiplex reactions were re-run as singular reaction mixes with the same primer concentration for the failed marker and additional MQH_2_0 replacing the successful marker primers to make up to 12µl. PCR products were diluted (S3) and either loaded singularly or combined into panels. To the ABI, 1µl of PCR product dilution and 10µl of loading mix was added. In cases of combined panels, 0.5µl of each PCR product was added to a sum of 1µl. Loading mix comprised Hi-Di™ Formamide (Applied Biosystems, 4311320) and GeneScan™ 500 ROX™ dye Size Standard (Applied Biosystems, 4310361) in a ratio of 400:1.

| S2: Sample location information | | | | | | | |
| --- | --- | --- | --- | --- | --- | --- | --- |
| Sampling site | Site code | Subbasin | Region | Country | Year | No. of thalli genotyped | |
|  |  |  |  |  |  | Free-living | Attached |
| AS1 | AS | Northern Baltic proper | Askö | Sweden | 2017 | 50 | 50 |
| AS2 | AS | Northern Baltic proper | Askö | Sweden | 2017 | 50 | 50 |
| AS3 | AS | Northern Baltic proper | Askö | Sweden | 2017 | 50 | 49 |
| AS4 | AS | Northern Baltic proper | Askö | Sweden | 2017 | 50 | 50 |
| AS5 | AS | Northern Baltic proper | Askö | Sweden | 2017 | 50 | 50 |
| AS6 | AS | Northern Baltic proper | Askö | Sweden | 2017 | 49 | 50 |
| SE1 | SE | Gulf of Finland | Seili | Finland | 2018 | 30 | 59 |
| SE2 | SE | Gulf of Finland | Seili | Finland | 2018 | 30 | 30 |
| TZ1 | TZ | Gulf of Finland | Tvärminne | Finland | 2017 | 30 | 30 |
| TZ2 | TZ | Gulf of Finland | Tvärminne | Finland | 2017 | 30 | 30 |
| TZ3 | TZ | Gulf of Finland | Tvärminne | Finland | 2017 | 30 | 30 |
| TZ4 | TZ | Gulf of Finland | Tvärminne | Finland | 2017 | 30 | 30 |
| TZ5 | TZ | Gulf of Finland | Tvärminne | Finland | 2018 | 35 | 26 |
| TZ6 | TZ | Gulf of Finland | Tvärminne | Finland | 2017 | 0 | 68 |
| TZ7 | TZ | Gulf of Finland | Tvärminne | Finland | 2017 | 0 | 77 |
| TZ8 | TZ | Gulf of Finland | Tvärminne | Finland | 2017 | 0 | 74 |
| SA1 | SA | Gulf of Riga | Saaremaa | Estonia | 2018 | 45 | 0 |
| SA2 | SA | Gulf of Riga | Saaremaa | Estonia | 2018 | 35 | 0 |
| HS1 | HS | Arkona basin | Hiddensee | Germany | 2018 | 32 | 32 |
| HS2 | HS | Arkona basin | Hiddensee | Germany | 2018 | 0 | 32 |

| S3: Microsatellite marker characteristics and associated PCR conditions. Primer sequence and repeat array taken from the respective articles. | | | | | | | | | | | | |
| --- | --- | --- | --- | --- | --- | --- | --- | --- | --- | --- | --- | --- |
| Locus | Source | Primer sequence (5’–3’) | Repeat array | Fluores-cent tag | T_a_ (^o^C) | PCR pg ^(‡)^ | Polymerase | Panels for PCR | Dilution | Preferred loading method | Panels for ABI | Number of alleles |
| L20 | Engel et al., (2003) | F- ACT CCA TGC TGC GAG ACT TC  R- CCT CGG TGA TCA GCA ATC AT | CTGG(CTG)8(TTG)3CTT(CTG)2 | HEX | 55^(†)^ | (1) | OneTaq® Hot Start | L20 | 1:150; 1:200 | Singularly | L94  L85  L20 | 26 |
| L58 | Engel et al., (2003) | F- AAA CGA AAA TGG CAC AGT GA  R- CCT TGC ATG TAG GAG GGA AC | (GA)19 | TAMRA | 52 | (3) | OneTaq® | L58 | 1:50 | Singularly | L58  FSP2 | 8 |
| L94 | Engel et al., (2003) | F- TTA GGA ATG GGC GGG ATG  R- GAT TTC GTG AGG CTG GTT CA | (GCA)3GACGAT(GCA)5ACA(GCA)5[GCT(VCA)6]2 | FAM | 55^(†)^ | (1) | OneTaq® Hot Start | L85  L94 | 1:75 | Panel | L94  L85  L20 | 19 |
| L38 | Engel et al., (2003) | F- TGC TAG CTG CTC TTG TGT GC  R- TAA CCT GTC GGT CGC AAC G | (GCT)11GCC(GVT)7 | FAM | 55 | (2) | OneTaq® | L38  FSP1  FSP3 | 1:120 | Panel | L38  FSP1  FSP3 | 15 |
| L85 | Engel et al., (2003) | F-GCT GAG TTG CCT TAC CGA CA  R- TAG GAT GAT AGG GCC GGA TT | (GT)3CG(GT)8GCGT | TAMRA | 55^(†)^ | (1) | OneTaq® Hot Start | L85  L94 | 1:75 | Panel | L94 L85 L20 | 27 |
| FSP1 | Perrin et al., (2007) | F- TCA AAA GCC AGC AGG GGT G  R- TCT TCT GGG AGC TGT AAA ATA GTC | (AG)11 | HEX | 55 | (2) | OneTaq® | L38  FSP1  FSP3 | 1:120 | Panel | L38 FSP1 FSP3 | 16 |
| FSP2 | Perrin et al., (2007) | F- GCA TCT GGT GTC ATT CCT TGT TC  R- TTG TTT GAG TGC CAC CTT GC | (TC)6CT(TC)3G(CT)5 | FAM | 52 | (4) | OneTaq® | FSP2 | 1:100; 1:150 | Singularly | L58 FSP2 | 10 |
| FSP3 | Perrin et al., (2007) | F- TGG AGG CCC TCC ACA GCC  R- TGC ATT GTA TGT CCT GTC CC | (AG)12 | TAMRA | 55 | (2) | OneTaq® | L38 FSP1 FSP3 | 1:120 | Panel | L38 FSP1 FSP3 | 5 |
| ^(†)^ touchdown PCR -1^o^C/cycle until T_a_  ^(‡)^ PCR programs: (1) 5 cycles of touchdown 95 ^o^C 30s, 60-55 ^o^C 30s (-1^o^C\cycle), 72 ^o^C 30s followed by 35 cycles of 95 ^o^C 30 s , Ta 30s , 30s 72 ^o^C; (2) 40 cycles of 94 ^o^C 30s, Ta 40s, 72 ^o^C 30s; (3) 35 cycle of 94 ^o^C 30s, Ta 30s, 72 ^o^C 30s; (4) 35 cycles of 94 ^o^C 30s, Ta 40 s, 72 ^o^C 30s. All programs had an initial denaturation step of 95 ^o^C 5min and final extension step of 72 ^o^C 5min. | | | | | | | | | | | | |

| 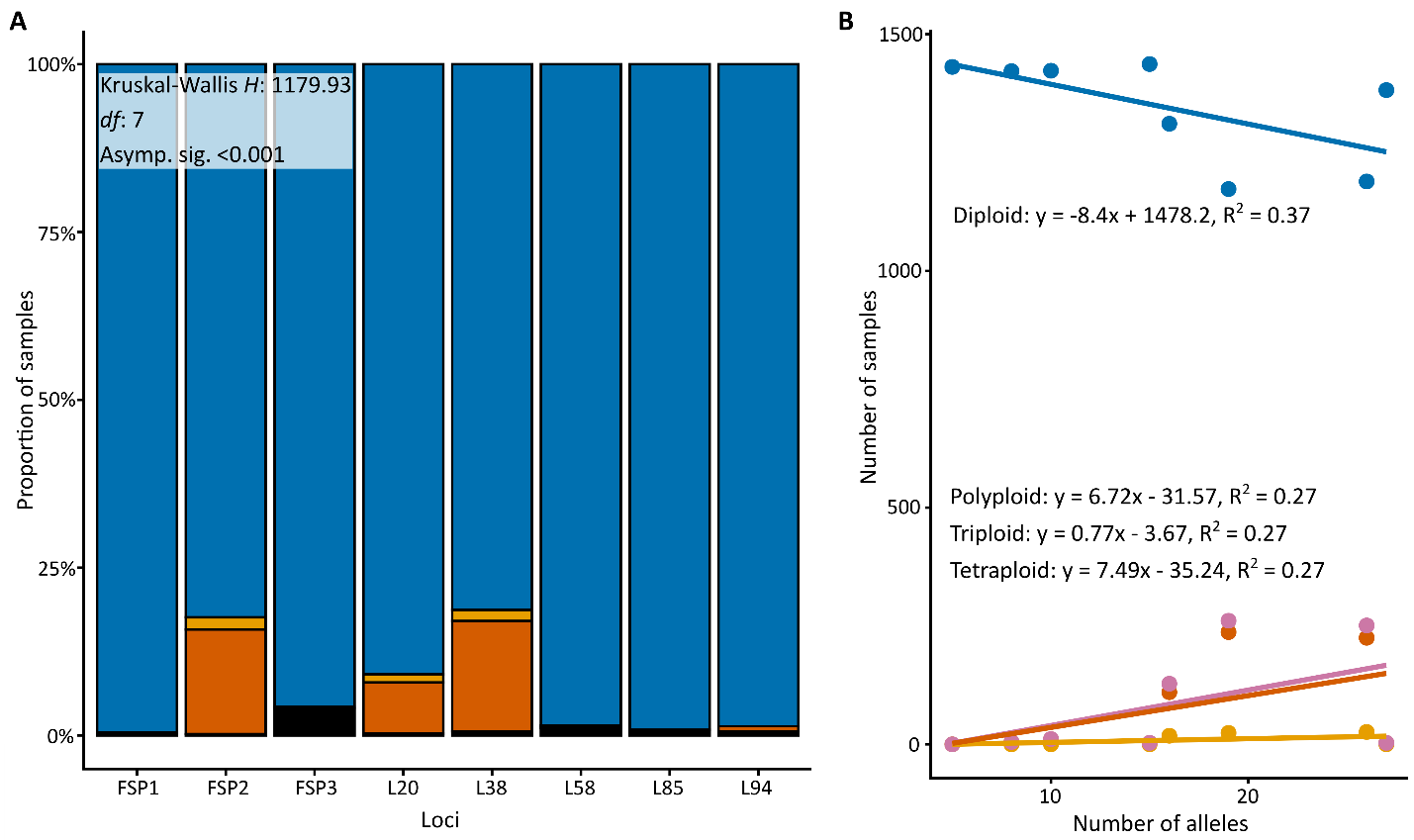 |
| --- |
| S4: Analysis of ploidy by loci determined by the number of alleles observed (≤2, diploid; ≥3, polyploid; 3, triploid; 4, tetraploid) in the microsatellite electropherograms. Observed ploidy levels across loci as a percentage of individuals at each loci (A) and trends in the determined ploidy level due to the allele variance of loci (B). Key: blue, diploid; orange, triploid; yellow, tetraploid; pink, all polyploids; black, missing data. |

| S5: Chi-square crosstabulation. Relationship between ploidy levels and the portion of multilocus genotype types.  Abbreviations: MLGs, multilocus genotypes. | | | | | | |
| --- | --- | --- | --- | --- | --- | --- |
|  |  | |  | MLGs | |  |
|  |  | |  | Unique | Clonal | Total |
| Ploidy | Diploidy | | Observed | 650 | 207 | 857 |
|  |  |  | Expected | 681.8 | 175.2 |  |
|  | Polyploid | | Observed | 498 | 88 | 586 |
|  |  |  | Expected | 466.2 | 119.8 |  |
|  | Total | | Observed | 1148 | 295 | 1443 |
|  | | | | | | |
|  | | Pearson Chi-Square | | | | 17.864 |
|  | | Asymptotic Significance (2-sided) | | | | <0.001 |

| S6: Probability of finding the observed clonal diversity under random mating (Corrected Nei’s diversity index) in clonal *Fucus* populations. Site abbreviations: A, attached; F, free-living; AS, Askö; HS, Hiddensee; SA, Saaremaa; SE, Seili; TZ, Tvärminne. | | | |
| --- | --- | --- | --- |
| Population | Observed diversity | Expected diversity | P value |
| AS1.F | 0.939 | 0.998 | 0.001 |
| AS2.F | 0.993 | 1 | 0.001 |
| AS3.A | 0.998 | 1 | 0.00 |
| AS3.F | 0.985 | 1 | 0.001 |
| AS4.F | 0.993 | 1 | 0.001 |
| AS5.F | 0.985 | 1 | 0.001 |
| AS6.A | 0.999 | 1 | 0.060 ^NS^ |
| AS6.F | 0.891 | 1 | 0.001 |
| HS.F | 0.948 | 0.999 | 0.001 |
| SA1.F | 0.805 | 1 | 0.001 |
| SA2.F | 0.936 | 1 | 0.001 |
| SE1.F | 0.989 | 1 | 0.001 |
| SE2.F | 0.995 | 1 | 0.002 |
| TZ1.F | 0.952 | 0.985 | 0.065 ^NS^ |
| TZ2.F | 0.844 | 0.999 | 0.001 |
| TZ3.F | 0.954 | 0.993 | 0.009 |
| TZ4.F | 0.869 | 0.999 | 0.001 |
| TZ5.F | 0.976 | 1 | 0.001 |
| TZ8.A | 1 | 1 | 0.054 ^NS^ |
| Overall | 0.999 | 1 | 0.001 |

| 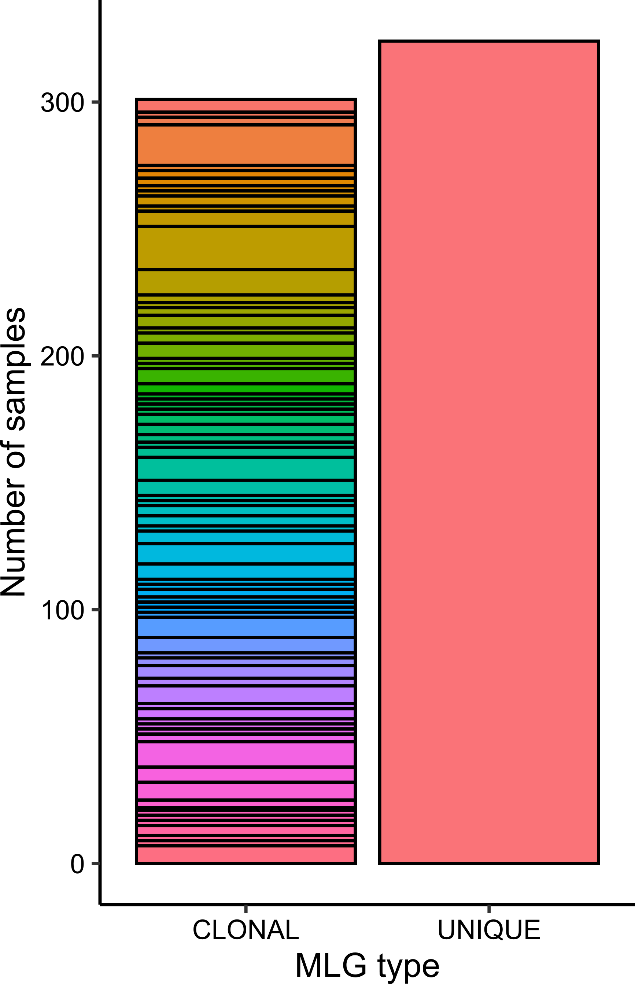 |
| --- |
| S7: Representation of multi-locus genotypes (MLGs) observed within the whole free-living population. Separated sections represent a single genet in the clonal MLG type. All samples within the unique MLG type represent singular genotypes. Colours portray no specific information. A total of 10 shared and 42 site specific clonal MLGs were observed. Mean number of ramets per genet = 4. |

| S8: Mann-Whitney Tests determining the differences in amounts of total multilocus genotypes (MLGs) (A) and clonal MLGs (B) between free-living and attached population. | | | |
| --- | --- | --- | --- |
| A | | | |
| Form | *n* | Mean Rank | Sum of Ranks |
| Attached | 18 | 23.58 | 424.50 |
| Free-living | 16 | 10.66 | 170.50 |
|  | | | |
| Mann-Whitney *U* | | | 34.50 |
| Wilcoxon *W* | | | 170.50 |
| Asymp. Sig. (2-tailed) | | | <0.001 |
| B | | |  |
| Form | *n* | Mean Rank | Sum of Ranks |
| Attached | 18 | 9.58 | 172.50 |
| Free-living | 16 | 26.41 | 422.50 |
|  |  |  |  |
| Mann-Whitney *U* | | | 1.50 |
| Wilcoxon *W* | | | 172.50 |
| Asymp. Sig. (2-tailed) | | | <0.001 |

| 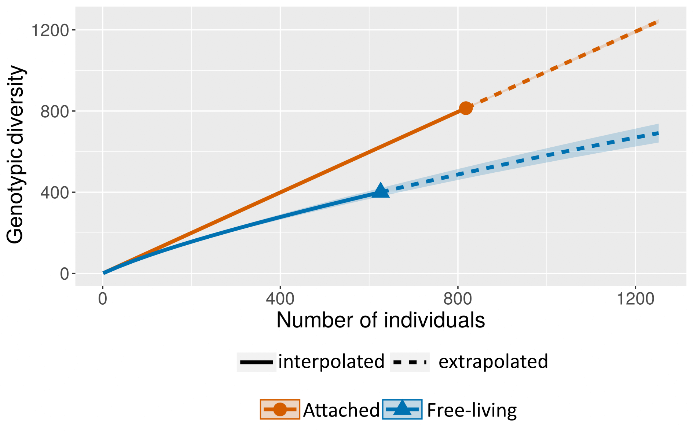 |
| --- |
| S9: Rarefaction analysis defining the genotypic diversity of both forms. |
